# Infantile Systemic Hyalinosis: A Case Report and Literature Review

**DOI:** 10.7759/cureus.46519

**Published:** 2023-10-05

**Authors:** Samah E Mohammed, Mohaned M Mohammed, Muhammad Saeed, Daifulah AL Zahrani, Badriah G Alasmari

**Affiliations:** 1 Pediatric Medicine, Armed Forces Hospital Southern Region, Khamis Mushait, SAU; 2 Pediatric Neurology, Armed Forces Hospital Southern Region, Khamis Mushait, SAU; 3 Pediatrics, King Saud Bin Abdulaziz University for Health Sciences, Jeddah, SAU; 4 Pediatrics, Armed Forces Hospital Southern Region, Khamis Mushait, SAU

**Keywords:** antxr2 gene, consanguinity, skin lesions, joint contractures, infantile systemic hyalinosis

## Abstract

Infantile systemic hyalinosis (ISH) is a very rare disorder belonging to the heterozygous group of genetic fibromatosis. There is a diffuse deposition of hyaline material in the skin, gastrointestinal tract, muscle, lymph node, spleen, thyroid, and adrenal gland due to which it presents clinically with multiple subcutaneous skin nodules, gingival hypertrophy, osteopenia, joint contractures, failure to thrive, and diarrhea with protein-losing enteropathy, and is associated with recurrent infections. The disease is caused by mutations in ANTXR2 also known as the CMG2 gene, which encodes the transmembrane-extracellular matrix assembly.

In this report, we describe a nine-month-old male diagnosed with ISH based on the clinical presentation of severe skin lesions, painful joint contractures, diarrhea, and failure to thrive. His diagnosis was confirmed by molecular DNA sequencing of the ANTXR2 gene. Consanguinity and molecular diagnosis will be helpful for early diagnosis and accurate management.

## Introduction

Infantile systemic hyalinosis (ISH) is a rare, inherited connective tissue disorder belonging to the heterogeneous group of genetic fibromatosis [1] characterized by progressive joint contracture, skin abnormality, severe chronic pain widespread hyaline in many tissues such as the skin, skeletal muscles, cardiac muscles, gastrointestinal lymph nodes, spleen, and thyroid. The first report on this was by Murray in 1873 as ''molluscum fibrosum'' [2] and then on juvenile hyaline fibromatosis (JHF) and (ISH) by Drescher et al. (1967) [3]. Two types of the disease are recognized in the medical literature: ISH and JHF [4]. Both conditions are characterized by painful joint contractures, gingival hypertrophy, and subcutaneous and perianal fleshy nodules [5].

However, ISH is distinguished by an earlier onset, more progressive and severe course, and death in early childhood. Nonetheless, there are striking histological similarities between the two subtypes. It has been suggested that both diseases represent different expressions of the same disorders. Recently, this has been confirmed by identifying mutations in the capillary morphogenesis protein 2 (GMC2) causing both JHF and ISH [6].

Cases of systemic hyaline fibromatosis have been described from almost all parts of the world but are most frequent among Arabs, Japanese, and Indians. Currently, the diagnosis depends on clinical presentation and genetic investigations. No specific treatment options are available, only supportive management is given [7].

Here, we report a new case from the southern region of Saudi Arabia who presented with massive skin lesions, generalized joint contractures, and failure to thrive [8]. We present this case with a typical clinical picture of infantile systemic hyalinosis for physicians to be more aware of the disease and diagnose it as early as possible.

## Case presentation

We present a nine-month-old male child delivered to first-degree consanguineous parents. He was the second baby; the first one is alive and well. The family history was negative for congenital malformations or genetic diseases.

**Figure 1:**
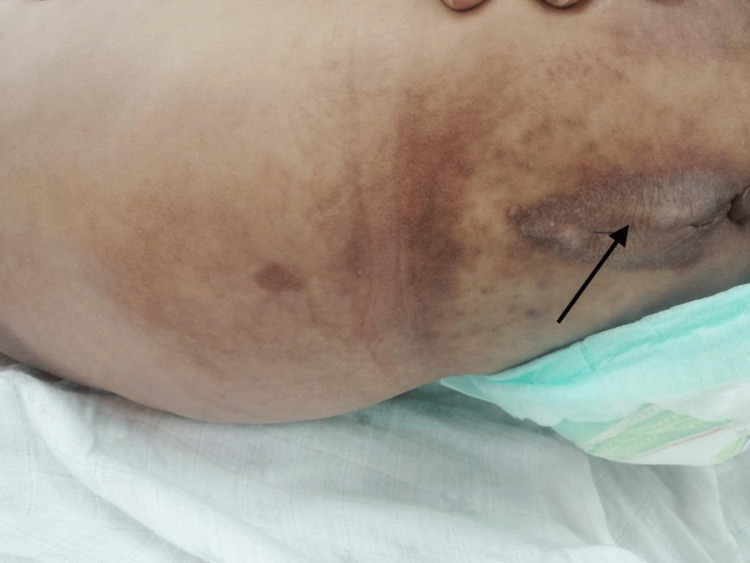
Hyperpigmented fleshy lesions located peri-anally

**Figure 2:**
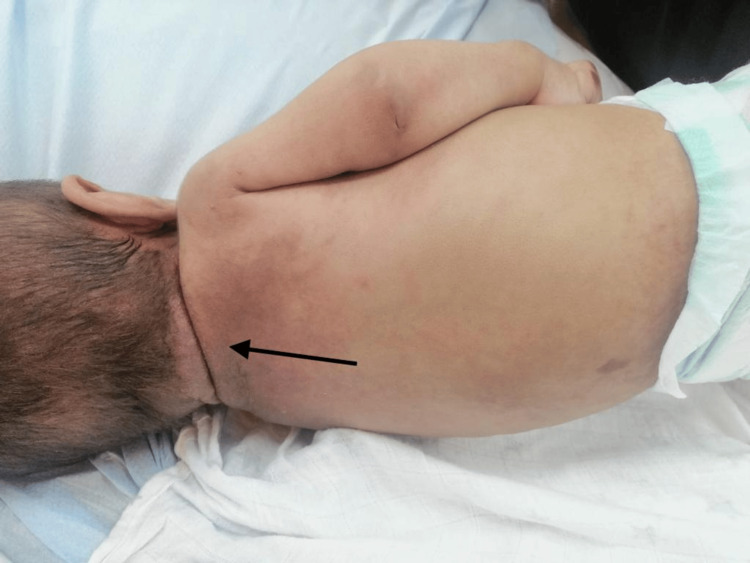
Hyperpigmentation in the upper back

**Figure 3:**
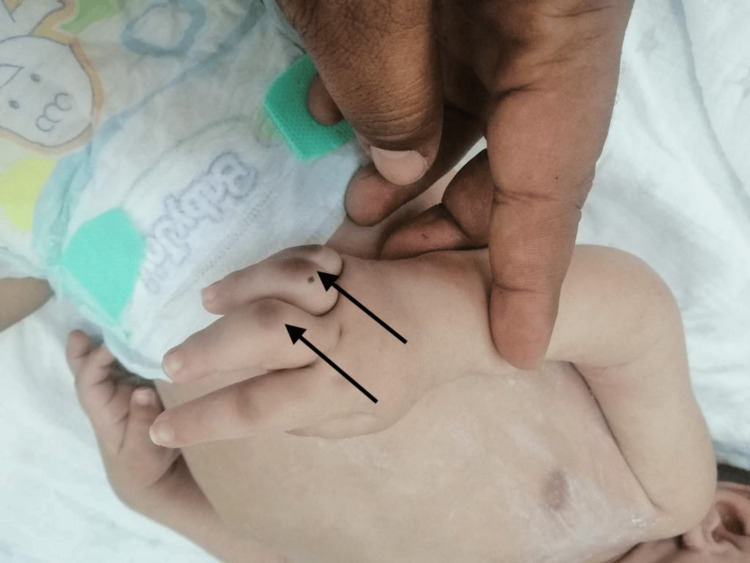
The picture depicts small joint contractures seen in the third and fourth proximal intraphalangeal joints

**Figure 4:**
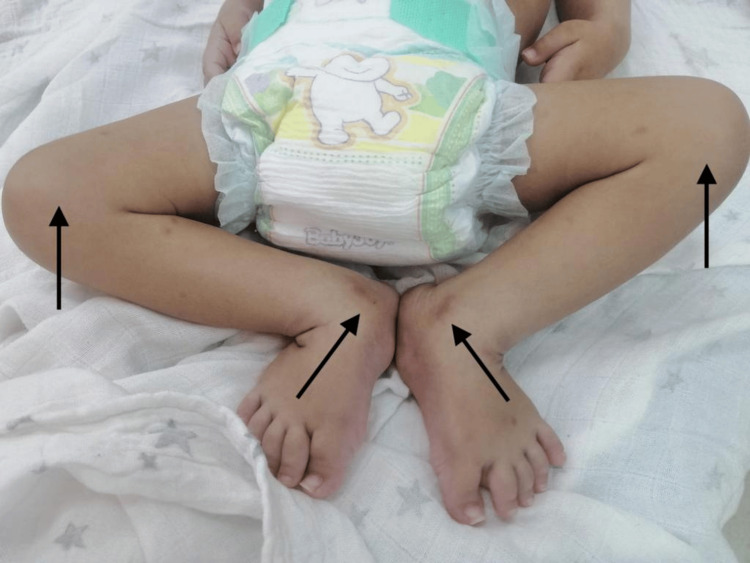
Bilateral knee and ankle joint contracture

He was a full-term baby born via emergency cesarean section due to fetal distress in cardiotocography (CTG) with decreased fetal movement observed by his mother but an otherwise uneventful pregnancy. His birth weight was 3.1 kg, and he was discharged from the hospital in stable condition. During the first month of life, the mother noticed pain during minimal handling of their child with limitation of movement of joints while changing the child's clothing, with severe pain on handling and progressive contractures and skin darkness with perianal masses. At the age of one month, he was admitted to the hospital with respiratory syncytial virus (RSV) bronchiolitis. Physical examination revealed a weight of 2.8 kg, dysmorphic features in the form of a broad forehead, gingival hypertrophy, and low-set ears. On examination of the skin, there was hyper-pigmentation of the skin around the mouth, the posterior lower part of the neck, different sites of both the upper and lower limbs, and anus not associated with focal nodules. Skeletal examination showed there were joint contractures of both upper and lower limbs involving even the small joints. Limited joint movement with pain and tenderness was noticed during the examination of the patient. There were fleshy perianal nodules on examination as seen in Figures 1-4.

Hematological and biochemical investigations were normal. A skeletal survey demonstrated generalized osteopenia. Ultrasound of the abdomen was normal. Ophthalmological examination was also normal. The diagnosis was ultimately confirmed by molecular genetic analysis of whole exome sequencing (WES), which revealed a homozygous pathogenic variant in the ANTXR2 gene, and correlating with the phenotype of the patient, a genetic diagnosis of hyaline fibromatosis syndrome was confirmed (Table 1).

**Table 1:** Whole exome sequencing for the patient Mendelian inheritance in man (MIM), Minor allele frequency (MAF), the Genome Aggregation Database (gnomAD), Autosomal recessive (AR), Homozygous (Hom.), Chromosome 4 (chr4)

GENE (Isoform)	Phenotype MIM	Variant	Zygosity	MAF gnomAD (%)	Classification
ANTXR2 (NM_058172.6)	228600 (AR)	c.134 T>C p.(Leu45Pro) chr4:80993581	Hom.	0	pathogenic

## Discussion

ISH presents in early life with growth failure, painful reduced movements of the limbs with joint contractures, osteoporosis with nodular thickening of the skin, gingival hyperplasia, severe chronic diarrhea, multiple sepsis, and ultimately death (Table 2) [9]. The clinical presentation is similar for ISH and JHF. Both diseases occur due to defects in the anthrax toxin receptor 2 (ANTXR2 gene). The clinical features of both include pain on minimal handling of the child. Progressive joint contractures of both upper and lower limbs involving small joints, gingival hypertrophy, and subcutaneous and perianal fleshy nodules share similar histological findings [5]. It is difficult to distinguish between them, but ISH presents early with severe and progressive forms that lead to death within the first two years of life and perhaps the development of malabsorption. Both diagnoses need a high index of suspicion.

**Table 2:** The diagnostic criteria including genetic variants and symptoms that were seen in our patient

Gene mutation	Variant	Symptoms (appears at birth or a few months later)	
ANTXR2 (NM_058172.6)	c.134 T>C p.(Leu45Pro) chr4:80993581	Musculoskeletal	Severe pain causing reduced spontaneous movement.
			Progressive joint contractures.
			Osteopenia.
		Gastrointestinal	Gastrointestinal symptoms (refractory diarrhea).
			Protein-losing enteropathy.
			Gingival hypertrophy.
		Dermatological	Hyperpigmentation over joints and bony prominence (over the Neck, Scalp, and Face).
			Fleshy nodules (perianally).
			Progressive skin thickening.
		Infectious disease	Recurrent infections.

Almost all characteristic clinical features of ISH were detected in our patient. After the diagnosis of the patient at the age of nine months, supportive management was initiated, including physiotherapy and nutrition support with multidisciplinary team follow-up. The patient developed fever, diarrhea, hypotension, and tachycardia. The patient required pediatric intensive care unit (PICU) admission where the patient was managed for septic shock and multiorgan failure with fluids resuscitation, inotropic support, and covered with antibiotic vancomycin and meropenem. The patient was managed by a multidisciplinary team, including PICU doctors, gastroenterologists, geneticists, nutritionists, infectious disease doctors, dermatologists, and social workers. Unfortunately, the patient died at the age of 10 months due to the worsening course of the disease.

ISH was reported globally in all ethnic groups and has a high prevalence among Middle Eastern and North African populations. ISH has a very poor prognosis, causing death within the first two years of life, with recurrent chest infections due to impaired chest wall movement as the leading cause of death [7]. There is no specific treatment, only supportive management. Few reports have documented the use of D-penicillamine with some improvement in joint mobility as a result of the inhibitory effect on collagen mutation [10]. Troublesome nodules can be surgically excised. Joint contractures needed dedicated physiotherapy staff. Systemic hyalinosis is still a less-understood entity of ground substance biology and poses diagnostic as well as therapeutic dilemmas for the treating physicians.

## Conclusions

Infantile systemic hyalinosis is a progressive disease causing death, and the numbers are rising in the world. The diagnosis of ISH requires full attention to clinical features. We suggest screening tools and early diagnosis because the child is normal at birth and shows the features of the disease later on in life. The establishment of guidelines for ISH would help make the diagnosis easier.
